# Improving the wellbeing and resilience of health services staff via psychological skills training

**DOI:** 10.1186/s13104-018-4034-x

**Published:** 2018-12-22

**Authors:** Joep van Agteren, Matthew Iasiello, Laura Lo

**Affiliations:** 1 Wellbeing and Resilience Centre, South Australian Health and Medical Research Institute, 5000 North Terrace, Adelaide, Australia; 2 Flinders Human Behaviour and Health Research Unit, College of Medicine and Public Health, Flinders University, Adelaide, Australia; 3 College of Nursing and Health Sciences, Flinders University, Adelaide, Australia

**Keywords:** Resilience intervention, Positive mental health, Psychological skills training, Wellbeing, Positive psychology intervention, Wellbeing and resilience program, Resilience

## Abstract

**Objective:**

Health services staff work in a stressful environment, which can negatively impact their mental health and wellbeing, and as a result can affect psychosocial and professional functioning. The implementation of resilience training aims to provide staff with basic psychological skills to improve mental health outcomes. The aim of the current pre-post study was to determine the short-term effects of group-based resilience training on clinical and non-clinical medical staff’s (n = 40) mental health outcomes.

**Results:**

The study showed statistically significant improvements in resilience (*r *= 0.51, *p *= 0.02) and wellbeing (*d *= 0.29, *p *= 0.001) from before to 1 month after the training. Participants with the lowest wellbeing and resilience scores at start of the training showed higher effect sizes compared to those with highest wellbeing and resilience scores, (*r *= 0.67 compared to *r *= − 0.36 for wellbeing scores and *d *= 0.92 compared to *d *= 0.24 for resilience scores); differences that point to particular impact of the training for people with the lowest baseline values. No significant changes in psychological distress as a result of depression, anxiety and stress were found. Brief implications of the findings for mental health and wellbeing interventions in the health services are discussed.

## Introduction

Health services staff, both clinical and non-clinical, operate in high stress environments, which are often under-resourced and under strain. This environment negatively impacts their mental health and wellbeing, with a substantial body of literature indicating high levels of stress and burnout, indicators of low wellbeing, as well as more serious symptoms pointing to mental illness in this occupational group [1–8]. The adverse consequences of stress, burnout, overall low wellbeing and mental health on individuals are well known and include diminished physical and psychosocial functioning [9, 10]. Additionally, these outcomes can result in higher rates of absenteeism [11] and when on the job, can lead to reduced professional functioning and presenteeism [12]. This in turn can lead to lowered quality of care [13] for patients that receive treatment by health professionals with low wellbeing and mental health issues. For instance, major medical errors, a leading cause of preventable death [14], increase with higher rates of burnout and depressive symptoms [7].

Pro-actively addressing positive mental health, being defined as high levels of wellbeing and the ability to function fully [15, 16], by providing basic psychological skills training to health services staff, is an intervention health service organisations can implement as a primary prevention strategy for the general workforce staff [17]. Furthermore, they can implement it as a targeted intervention focusing on staff who display low levels of wellbeing and resilience (hereafter referred to as low baseline wellbeing and resilience), as this population is at most risk of developing mental illness in the future [18, 19].

The current study aimed to establish the short-term effects of group-based resilience training on mental health outcomes, with the purpose of establishing local baseline data and determining preliminary effectiveness in changing wellbeing and resilience, and indicators of mental distress due to depressive symptoms, anxiety and stress. A secondary focus was determining the effect of baseline wellbeing and resilience scores on effect sizes of each respective outcome. The training was hypothesized to lead to improvements in wellbeing and resilience, and reductions in mental distress, with results expected to be particularly profound for those with lowest levels of baseline wellbeing and resilience.

## Main text

### Methods

#### Participants and setting

Participants were clinical and non-clinical staff working within a major public healthcare provider in Adelaide, Australia. Adult (18+) participants could self-select or were appointed by managers to participate in a resilience project. The project consisted of 2 days of resilience training, delivered in three groups of between 50 and 60 participants facilitated by three professional trainers. Each participant was given a positive mental health assessment prior to and 1 month after the training, resulting in an individual personalised report, which was provided to the participant immediately after completing the assessment. One hundred and sixty staff undertook the training, of which 40 staff (25%) provided consent to study their training data and provided data for two measurement time-points (baseline and 1 month after the training).

The resilience training was an adaptation of the TechWerks Resilience Training Program (http://www.technologywerks.com and http://www.4-9-north.com) and was delivered by experienced professional trainers. The training consisted of 10 skills (see Table 1) originating from best-practice positive psychology approaches and evidence-based methods for improving wellbeing and resilience [20, 21]. The impact of the intervention has been successfully demonstrated in a range of settings including with workers on the brink of retrenchment and older aged carers [22].

**Table 1:** Overview of 10 skills taught in the resilience training

Meaning making	Learn to cognitively appraise challenges and failures in a healthy and productive way through a focus on meaning
Event-thought-reaction connections	Increase awareness of how thoughts drive reactions to events, and determine if thoughts and reactions are helping individuals work towards their goals, act upon their values, improve their performance and strengthen their relationships
What’s most important	Increase individual awareness of what influences unproductive reactions (emotional and/or physical) that may interfere with their performance, goals or relationships
Balance your thinking	Help individuals cognitively appraise situations in an accurate manner that is based upon evidence
Cultivating gratitude	Build optimism, positive emotions and resilience by bringing ongoing attention to gratitude as a cognitive process
Mindfulness	Teach individuals to regulate their attention in a focused, open and non-judgemental manner
Interpersonal problem solving	Teach individuals the elements to address interpersonal problems in a respectful manner with healthy and productive emotional expression, and use of compromise
Active constructive responding	Increase awareness of communication patterns and responses that maintain, strengthen, and cultivate positive and important relationships
Capitalising on strengths	Increase individual awareness of theirs and others personal strengths, and how to apply strengths across all life domains
Values based goals	Increases individual awareness of their values, and how to translate these values into actions and goals

The PERMA-profiler [23] was used to measure wellbeing. It is a validated measurement of overall wellbeing or flourishing, and correlates highly with other measures of subjective wellbeing and life satisfaction [24]. Resilience was assessed using the Brief Resilience Scale (BRS) [25], which measures the outcome of being resilient to stressful events; a focus that makes the tool different from measures that capture resilience as a trait, or capture the resources required for resilience to occur [26]. Scores that are lower than 3.00 on the BRS indicate low resilience, while scores between 3.00 and 4.30 indicate normal resilience. Scores higher than 4.30 indicate high resilience. Mental distress as a result of mood problems, anxious feelings and stress was measured using the Depression Anxiety Stress Scale 21 item (DASS-21) [27, 28]. The DASS-21 is a widely used screening tool for mental distress, which has widely accepted cut-off scores per domain, where normal ranges are indicated by scores up to 9 for depression, 7 for anxiety and 14 for stress, with higher scores indicating potential disorder ranging from mild and moderate to severe and extremely severe.

Statistical analyses were performed using IBM SPSS 25. Depending on the data distribution, paired t-tests or Wilcoxon signed-rank tests being performed to determine between time differences. Effect sizes were estimated using Cohen’s *d* for parametric techniques, and *r* for non-parametric distributions, where Cohen’s rules of thumb for interpretation of the effect sizes were used; small effect *d *= 0.2 and *r *= 0.1, medium effect *d *= 0.5 and *r *= 0.3, large effect *d *= .8 and *r *= 0.5 [29]. To assess the influence of baseline differences on training effectiveness, scores were dichotomised. For resilience the official cut-off criteria to demonstrate “low resilience’ of scores less than 3.00 were used to place participants into the low resilience group, with all other scores being placed in the high resilience group. As the PERMA-profiler does not come with cut-off scores, participants were divided into high and low baseline wellbeing by using the median wellbeing scores at baseline.

### Results

Forty participants with a mean age of 44.68 (sd = 9.83) were included in the study. Thirty out of forty (75%) participants were female, with the majority of the sample (65%) having a college, university or post-graduate qualification.

The study found statistically significant improvements in wellbeing (*p *= 0.001) and resilience (*p *= 0.02), with small to moderate effect sizes found for the overall sample, see Table 2 for pre- and post-scores, and effect sizes. After dichotomising baseline wellbeing scores, those with low median baseline wellbeing scores demonstrated higher effect sizes for wellbeing (*r *= 0.67) compared to those with high baseline wellbeing scores (*r *= − 0.36). Similarly, those with low baseline resilience scores demonstrated higher effect sizes for resilience from pre to post training (*d *= 0.92), compared to those with high baseline resilience scores (d = 0.24).

**Table 2:** Outcome data for main mental health parameters pre and post (1-month) intervention for all participants (n = 40)

Variables	Baseline				1 month follow-up				p	*d*/*r*
	Mean	SD	Median	IQR	Mean	SD	Median	IQR		
Wellbeing	7.37	1.13	7.63	1.53	7.74	1.27	7.97	1.38	0.001**	*r *= 0.51
Resilience	3.41	0.88	3.50	1.35	3.67	0.91	3.83	1.40	0.02*	*d *= 0.29
Depression	4.80	6.97	2.00	6.00	4.95	8.09	2.00	6.00	0.82	*r *= 0.04
Anxiety	4.50	6.19	2.00	4.00	4.35	5.94	4.00	6.00	0.95	*r *= 0.01
Stress	10.80	7.55	10.00	10.00	9.35	7.95	4.00	10.00	0.14	*r *= 0.24

*IQR* interquartile range, *SD* standard deviation, *d* Cohen’s d (effect size estimate for variables with a parametric distribution), *r* correlation (effect size estimate for variables with a nonparametric distribution)

* Significant at p = 0.05 level

** Significant at p = 0.01 level

For the overall sample, no significant improvements in mental distress due to mood problems, anxiety and stress were found. There were only a low number of participants demonstrating baseline distress values that would enable change to be detected. Only eight out of 40 participants reached the threshold for mild mental distress due to mood problems, nine for anxiety problems, and eleven for stress. This was particularly the case for depression and anxiety scores, with the majority of participants scoring a zero or two, the two lowest possible scores, for depression (68%) and anxiety (55%).

### Discussion

The current study found significant positive effects of group-based training on wellbeing and resilience for general (clinical and non-clinical) staff working in the medical sector, particularly for those with the lowest baseline values. The large majority of participants in this study demonstrated no baseline mental distress values that were susceptible to change, thereby leading to an inability to determine the impact of the training on mental distress.

Improving the wellbeing of health services staff can be beneficial for both staff and patient, as poor wellbeing is associated with reduced clinical care capacity [13, 30], and is a risk factor for developing mental illness in the short and long-term [19, 31, 32]. Capacity-building interventions such as the one studied here are particularly effective for those with lower levels of wellbeing, and results found by this study strengthen the argument that they should be considered as options to strengthen the mental health and wellbeing of health professionals, either as a preventative solution, or as a method to reach health professionals who are at-risk. This is particularly important in light of the challenges and stigma surrounding mental illness help-seeking in the medical sector [3, 33–35]. Despite methodological limitations (e.g. lack of a randomised controlled design, low sample size), the current study’s positive results strengthen the existing evidence for the utility of positive mental health interventions for health services staff [36], and provide useful insights into local baseline data.

#### Limitations

- The lack of a randomised controlled design prevents the cause-effect relation between the wellbeing and resilience intervention and mental health outcomes to be made.
- Only short-term effects were studied, there is a need for long-term follow-ups.
- The majority of participants did not show high enough psychological distress symptoms to positively change, making it impossible to determine the potential impact of the training on indicators of psychological distress in this study.
- The absence of information regarding staff’s role within the organisation prevents generalisations from being made to specific roles (e.g. are nurses more receptive to training than psychologists).
- Using the median-split to create categories of ‘high’ and ‘low’ wellbeing, in the absence of clear cut-off scores, is a limitation and warrants caution when interpreting the findings.
